# Immunohistochemistry and molecular biology studies of apelin and apelin receptor in queen placenta

**DOI:** 10.1007/s11259-025-10766-0

**Published:** 2025-05-20

**Authors:** Sara Pastore, Cecilia Dall’Aglio, Margherita Maranesi, Guillaume Robiteau, Viola Zappone, Tiziana Caspanello, Alain Fontbonne, Angela Polisca, Andrea Verini Supplizi, Alessandro Troisi

**Affiliations:** 1 Department of Veterinary Medicine, University of Perugia, Via San Costanzo 4, Perugia, 06126 Italy; 2 Centre d’Etude de Reproduction des Carnivores (CERCA), École Nationale Vetérinaire d’Alfort, 7 Av. du Général de Gaulle, Maisons-Alfort, Francia Paris, 94700 France; 3 Department of Veterinary Medicine, University of Messina, Polo Universitario Annunziata, Messina, 98168 Italy; 4 School of Biosciences and Veterinary Medicine, University of Camerino, Via Circonvallazione 93/95, Macerata, 62024 Italy

**Keywords:** Adipokines, Adipose tissue, Cat, APJ receptor, Pregnancy

## Abstract

Placenta is a tissue where vasculogenesis, blood pressure and blood flow are dramatically important to allow normal embryonic and foetal growth and requires the production of numerous growth factors, hormones and transcription factors. Apelin is a pleiotropic peptide, and its major action relates to energy metabolism, cardiovascular function, body fluid homeostasis via its receptor. The involvement of the apelinergic system during pregnancy in veterinary medicine has been investigated only in bitches. Thereafter, the aim of our study was to investigate, for the first time, presence and distribution of this system in the queen placenta at mid- and end-gestation. Ten pregnant mixed-breed queens were used. The animals were equally divided into two groups based on the stage of pregnancy (mid and end gestation) and, with the written consent of their owners, were subjected to ovariohysterectomy or non-conservative caesarean section. The Real-Time PCR (RT-PCR) analysis showed the presence of transcripts for apelin and its receptor in all the foetal and maternal placenta samples processed. The immunohistochemical (IHC) study evidenced the presence and the distribution of positive immunoreactions for apelin and its receptor in all the samples observed. In particular, in the placental labyrinthic portion, apelin and apelin receptor immunopositivity was evident in the cytoplasm of trophoblasts and endothelial cells. The uterine glands also exhibited a positive immune reaction for apelin and corresponding receptor. Based on our results, apelin and its receptor, also in the queen placenta, could be an important system involved in the physiological development of placenta, embryo and foetal growth.

## Introduction

Apelin (also known as APLN), a 36 amino acid peptide originally identified from the bovine stomach, is the natural ligand of the G protein-coupled receptor (also called APLNR) which has high structural homology with the type 1 angiotensin receptor (Tatemoto et al. 1998). Apelin and its corresponding receptor form the apelinergic system. Many studies reported its expression in different mammalian tissues (Carpene et al. 2007; Falcao-Pires et al. 2010; Medhurst et al. 2003; Mercati et al. 2018; O’Carroll et al. 2000). For this reason, it is possible to hypothesize that APLN is present in different mammalian tissues with multiple biological actions during physiological or pathological conditions. Depending on the cell type studied, the activation of APLNR results in the stimulation of numerous intracellular effectors such as extracellular signal-regulated kinase, protein kinase B (PKB or AKT), and p70S6 kinase in the inhibition of cAMP production (D’Aniello et al., 2009). Although progress has been made in recent years in clarifying the physiological significance of APLN molecule and receptor, much remains to be discovered about the expression of the apelinergic system and precisely how it affects several physiological functions (O’ Carroll et al., 2013).

The involvement of apelinergic system in the control of reproduction was described in human and veterinary medicine (Kurowska et al. 2018). In particular, this important system is expressed in the granulosa cell and corpus luteum (CL) of various animal species (Estienne et al., 2019; Gupta et al. 2023; Mercati et al. 2019; Pirino et al. 2017; Pope et al. 2012; Rak et al. 2017; Różycka et al. 2018; Shokrollahi et al. 2021, 2022, 2023, 2024A, 2024B). Therefore, it is possible to hypothesize a potential role of APLN in the control of several aspects of ovarian cell function such as folliculogenesis, steroid hormone secretion, proliferation or apoptosis (Kurowska et al. 2018).

During embryonic and foetal growth, the apelinergic system is mainly involved in angiogenesis via endothelial cells proliferation and assembly. Infact, APLN is required for normal vascular development in frog and mouse embryos (Cox et al. 2006). Furthermore, the apelinergic system is expressed at the foetal-placental interface and in numerous foetal tissues (Mayeur et al. 2016). The apelinergic system also plays an important role in vascular smooth muscle proliferation and pericytes permeability. In particular, APLN exerts vasomotor effects causing vasodilation or vasoconstriction. These dual actions are attributed to the presence of APLNR in endothelial and smooth muscle cell layers of the blood vessel but the mechanism underlying this dual activity is not yet clear (Mughal et al., 2018). Because of the cardiovascular protective role and neovascularization action of APLN in addition to its role in the regulation of fluid homeostasis and metabolic functions, the apelinergic system may be important for maternal adaptation during gestation (Van Mieghem et al. 2010). Pregnancy entails dramatic cardiovascular, body fluid and metabolic changes (Van Mieghem et al. 2010) necessary to support the increased vascular demand of the uterus, placenta and foetus (Van Mieghem et al. 2016). In this regard, the apelinergic system is an emerging target for the regulation of cardiovascular homeostasis (Japp and Newby 2008). The placenta is a tissue where vasculogenesis, blood pressure, and flow are important to allow a normal embryo and foetal development and growth (Cobellis et al. 2007). The presence of APLN in human placenta tissue indicates its involvement in foetal development through a correct regulation of placenta formation (Cobellis et al. 2007).

In humans, Dawid et al. (2019) and Vaughan et al. (2019) reported that APLN stimulates trophoblast System A-mediated amino acid transport and plays an important role in controlling the production of steroid and protein hormones in placental trophoblastic BeWo cells respectively. During pregnancy, in veterinary medicine, the apelinergic system has been investigated and evidenced only in bitches (Troisi et al. 2020). Therefore, the aim of our study was to extend this research also in the queen placenta at mid- and end-gestation in order to verify APLN presence and distribution. The knowledge obtained will be useful in fully understanding the control of reproductive activity in normal and, subsequently, pathological conditions.

## Materials and methods

### Animals

Placental tissues were collected from 10 pregnant mixed-breed queens aged 2–5 years with an average weight of 3–5 kg. The animals were registered at the Teaching Hospital of the Department of Veterinary Medicine, University of Perugia, and were divided into two groups based on the stage of pregnancy: group 1 (mid-gestation: *n* = 5) and group 2 (end gestation: *n* = 5). Moreover, these animals, with the written consent of their owners, were subjected to ovariohysterectomy (group 1) or non-conservative caesarean section (group 2). The sample size was constrained by clinical availability. No animals were operated for the exclusive purpose of this study, otherwise the tissues examined would have undergone to disposal procedures. The experimental procedures were approved by the University Bioethics Committee of the University of Perugia, protocol number 114,630.

### Ultrasound examination

Pregnancy diagnosis was carried out by ultrasonography (Esaote My Lab 30; Genova, Italy) using a microconvex probe (5.5–7.5 MHz) for both bidimensional and color Doppler ultrasound scanning. The gestational periods were calculated based on the anamnestic data, (day of mating), and by ultrasound biometric parameters (Beccaglia et al. 2016; Michel et al. 2011).

### Surgical procedures

The ovariohysterectomy was carried out under general anaesthesia using the following protocol: premedication with methadone 0.2–0.4 mg/kg intramuscular (IM) or intravenous (IV) and medetomidine 1-5 mcg/kg; induction with preoxygenation and propofol 4–6 mg/kg IV; maintenance through isoflurane, adjusting the vaporiser setting according to anaesthetic depth + Ringer 10 ml/kg/h IV. Postoperative analgesia used buprenorphine 0.01–0.03 mg/kg IM. After two days of hospitalization, the queens were returned to their owners. The non-conservative caesarean section was carried out under general anaesthesia using the following protocol: induction with preoxygenation and propofol 4–6 mg/kg IV; maintenance through isoflurane, adjusting the vaporiser setting according to anaesthetic depth + Ringer 10 ml/kg/h IV. and, after removing the last kitten, methadone 0.2–0.4 mg/kg IV; finally, post-operative analgesia using buprenorphine 0.01–0.03 mg/kg IM.

### Tissue collection and processing

After ovariohysterectomy or caesarean section, placenta samples were promptly removed and thoroughly washed with saline. Within a few minutes, under stereoscopic magnification, tissue samples were quickly reduced and destined for subsequent examination.

For the molecular biological studies, the samples were rinsed with RNase-free water and then frozen at − 80 °C for later evaluation of gene expression. For the immunohistochemical analysis, tissue samples of placenta were fixed by immersion in 4% (w/v) formaldehyde in phosphate buffered saline (PBS) (pH 7.4) for 24 h at room temperature and subsequently processed following routine tissue preparation procedures (Dall’Aglio et al. 2014).

### Reagents

For molecular biology analysis, deoxyribonuclease I (DNAase I Amp. Grade), Superscript III Reverse Transcriptase (Superscript III First-Strand Synthesis System), and DNA ladders were obtained from Life Technologies Italia (Monza, Monza Brianza, Italy). Reagents for isolation and purification of total RNA (TRIzol), Taq DNA polymerase (Platinum), RNAse-free tubes, water and deoxyNTPs, and primers for APLN and APLNR were also acquired from Life Technologies. NucleoSpin Gel and PCR clean up were from Macherey-Nagel Inc (Bethlehem, PA, USA).

For immunohistochemical analysis, the rabbit polyclonal anti-APLN antibody (NBP2-31176) was from Novus Biologicals (Novus Biologicals, USA); the mouse monoclonal anti-APLNR antibody (sc-517300) was from Santa Cruz Biotechnology (Santa Cruz, CA, USA); the normal horse serum (s-2000) and the two secondary biotin-conjugated antibodies, horse anti-mouse (BA-2000) and horse anti-rabbit (BA-1100), as well as the Avidin-Biotin Complex (ABC Kit, PK-4000) and Diaminobenzidine (DAB, SK-4100) were from Vector Laboratories (Vector Laboratories, Burlingame, CA, USA). Finally, the Eukitt (03989) was from Sigma-Aldrich.

### RNA extraction and RT-PCR

Total RNA was extracted from placenta samples (30 mg each) as previously described (Maranesi et al. 2016). Five micrograms of total RNA were reverse transcribed in 20 µL of Superscript III First-Strand Synthesis System using random hexamer according to the protocol provided by the manufacturer.

Genomic DNA contamination was checked by developing the polymerase chain reaction without reverse transcriptase. The multiplex PCR amplification was performed as previously described (Maranesi et al. 2018; Troisi et al. 2020) with the use of 1.0 µL of complementary DNA as a template for APLN and APLNR primers (Table 1).

**Table 1:** Primers of *APLN* and *APLNR* used for RT-PCR

Gene	NCBI seq. ref.		Primers	Bp
*APLN*	XM_004000879.5	F	CCCCACCTCCTCCCATCTAA	104
		R	TGAGGCCCACTGAAGAAGG	
*APLNR*	XM_006937323.3	F	GGGCCAGCCAAGAGTTAACT	101
		R	TGGGATAACCTAGTGCCCGA	

Cycling conditions consisted of an initial denaturizing cycle at 94 C for 75 s followed by 35 cycles for each target gene at 94 C for 15 s, at 60 C for 30 s, at 72 C for 45 s, and a final extension step at 72 C for 10 min. Within each experiment, the complete set of samples was processed in parallel in a single PCR using aliquots of the same PCR master mix. The amplified PCR-generated products (18 µL of 25 µL total reaction volume) were analysed by electrophoresis on 2% agarose gel using ethidium bromide staining. The amplified products, collected from agarose gel after electrophoresis, were purified with NucleoSpin Gel and PCR clean-up and their identity confirmed by DNA sequencing using Sanger’s method.

### Immunohistochemistry

Samples were serially cut at a thickness of 5 μm. The histological sections were then collected on poly-L-lysine-coated glass slides, dewaxed, rehydrated and microwaved for 3 cycles of 5 min each at 750 W in citrate buffer (pH 6.0) to expose the epitopes to the antibodies. The sections were processed for immunohistochemical reaction following microwaving. All subsequent steps were carried out in a moist chamber at room temperature to prevent evaporation of the reagents. After a proper cooling, in order to prevent the non-specific binding of primary antibodies, the sections were treated with 3% hydrogen peroxide solution for 10 min to block endogenous peroxidase activity and then they were incubated for 30 min with normal horse serum (1:10 in PBS). Subsequently, each section was incubated overnight with one of the following primary antibodies at a dilution of 1:100: anti-APLN or anti-APLNR. The following day, after washing in PBS, the sections were incubated for 30 min with the corresponding secondary biotin-conjugated antibodies, horse anti-mouse IgG (for APLNR) or horse anti-rabbit IgG (for APLN), both at a dilution of 1:200, and they were then processed for 30 min using the Vectastain ABC kit. Subsequently, the tissue samples were repeatedly rinsed with PBS and developed with the chromogen solution. After several rinses in PBS, the sections were counterstained with haematoxylin, dehydrated and mounted in Eukitt.

Sections in which the primary antibodies were omitted were used as a control of unspecific staining. All tissue analyses were carried out on coded slides using a light microscope (Nikon Eclipse E800) connected to a digital camera (Dxm 1200 Nikon digital camera). For processing images, an image analysis system was used (Lucia, Laboratory Imaging Ltd.). The settings for image capture were standardized by subtracting the background signals obtained from the matched tissue sections which had not reacted with the primary antibodies and which were used as immunohistochemical controls (Dall’Aglio et al. 2012).

## Results

### Molecular biology

**Fig. 1:**
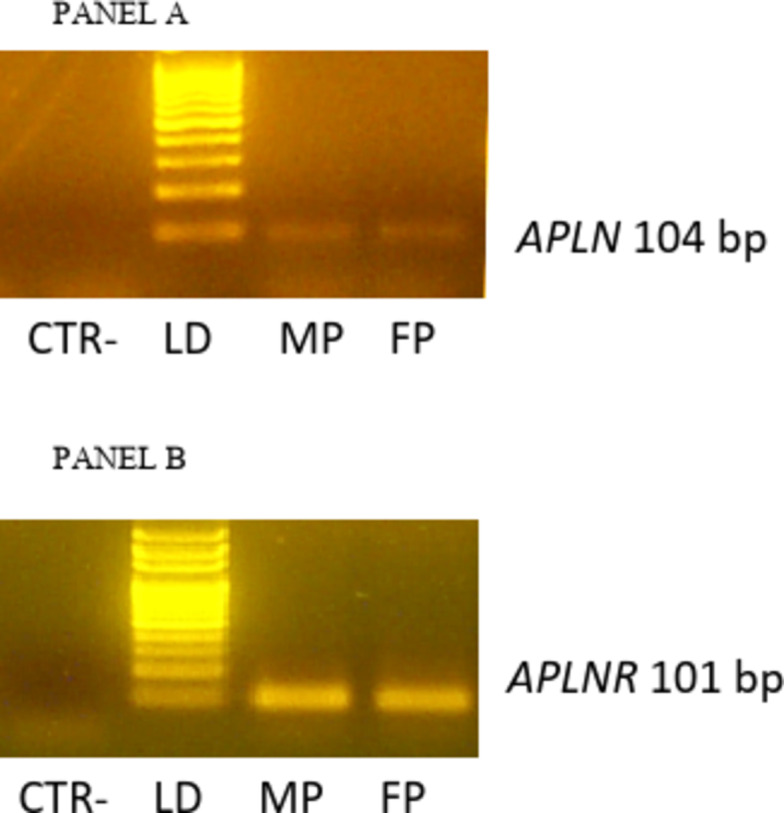
Gene expression of *APLN* (panel A) and *APLNR* (panel B) in maternal (MP) and foetal (FP) placenta of queens. Representative agarose gel electrophoresis stained with ethidium bromide to verify matching between expected and obtained PCR products. For every PCR, a negative control (CTR-) were included, LD = 100 bp DNAladder

The RT-PCR analysis showed the presence of transcripts for APLN and APLNR in all the samples processed of foetal and maternal placenta (Fig. 1).

### Immunohistochemistry

The immunohistochemical study evidenced the presence and the distribution of positive immunoreactions (IR) for APLN and APLNR in all the samples observed of both foetal and maternal placental portions, tested at mid and late pregnancy.

**Fig. 2:**
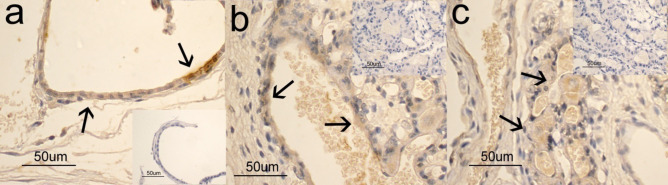
APLN immunohistochemical study in the feline placenta and uterus. In **a**) immunopositivity is localized in the cytoplasm of epithelial glandular cells (arrows); in **b**) immunopositivity is localized in the cytoplasm of endothelial cells and in blood capillaries (arrows); in **c**) immunopositivity is localized in some trophoblasts (arrows) of the placental labyrinthic portion

**Fig. 3:**
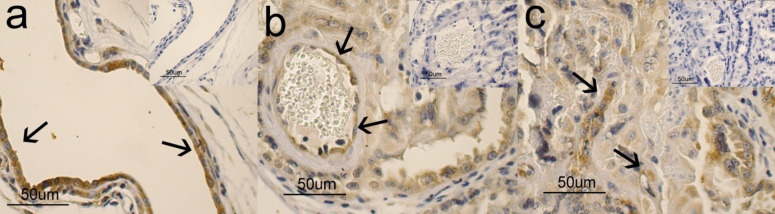
AAPLNRimmunohistochemical study in the feline placenta and uterus. In **a**) immunopositivity is localized in the cytoplasm of epithelial glandular cells (arrows); in **b**) immunopositivity is localized in the cytoplasm of endothelial cells (arrows), in blood capillaries; in **c**) immunopositivity is localized in some trophoblasts (arrows) of the placental labyrinthic portion

In particular, in the placental labyrinthic portion, APLN and APLNR immunopositivity was evident in the cytoplasm of trophoblasts and endothelial cells (APLN Fig. 2c and b; APLNR Fig. 3c and b). Variations in the intensity of immunolabeling for APLN and APLNR were observed between the different periods of pregnancy. However, given the prevalently qualitative nature of the immunohistochemical technique, they were not quantified. Moreover, even if not included into the structure of the placenta, the uterine glands also exhibited a positive IR for APLN and APLNR (Figs. 2a and 3a). Controls were always negative (see inserts in the figure).

## Discussion

Apelin is a pleiotropic peptide expressed in numerous tissues and therefore involved in many physiological processes. Its action is mediated by its receptor, APLNR, a transmembrane receptor associated with a G protein (Estienne et al., 2019). The major action of the apelinergic system is related to body fluid homeostasis, energy metabolism, cardiovascular function and reproductive function. During human gestation APLN is involved in pregnancy by (i) regulation of blood pressure and angiogenesis in the preeclampsia pathophysiology; (ii) effects on plasma volume expansion in foetal growth; and (iii) regulation of glucose metabolism in the gestational diabetes pathophysiology (Malamitsi-Puchner et al. 2007; Van Mieghem et al. 2010; Yamaleyeva et al. 2015). Recently in humans the capacity of APLN to stimulate trophoblast System A-mediated amino acid transport and to control the production of steroid and protein hormones in placental trophoblastic BeWo cells were reported by Dawid et al. (2019) and Vaughan et al. (2019) respectively. Mlyczyńska et al. (2020) noted that APLN is produced by trophoblastic cells and can be involved in the early human placental development.

Our results demonstrated, in accordance with those reported in humans (Cobellis et al. 2007; Furuya et al. 2012; Yamaleyeva et al. 2015) and bitches (Troisi et al. 2020), the apelinergic system transcript existence confirmed by the presence and the distribution of positive immunoreactions (IR) in all the samples examined, in both foetal and maternal placental portions. In particular, APLN and APLNR immunopositivity was evident in the cytoplasm of trophoblasts. Since the last cell type represents the proliferative population inside placental villi, it is possible to hypothesize that in the queen the direct role of the apelinergic system was on placenta cell proliferation, in accordance with what has been reported in “in vitro” studies in humans (Mlyczyńska et al. 2020).

Variations in the intensity of immunolabeling for APLN and APLNR observed between the different periods of pregnancy probably could reflect the expression of the corresponding antigens. However, since immunohistochemistry is an exquisitely qualitative method, it is not possible to use these observations as an expression of a variation in the amount of antigen resident. Further studies are in progress to verify if in the queen, as well as in humans (Cobellis et al. 2007), there is a modulation of apelinergic system throughout pregnancy.

Finally, in our study, the presence of APLN was also observed in endothelial cells of the placental blood capillaries at mid- and at end-gestation, in accordance with data reported in human (Mlyczyńska et al. 2020) and on the contrary to what was observed in bitches (Troisi et al. 2020). The discrepancy in the distribution of APLN in the reported animal species could reflect an interspecies difference that has already been called into question for other molecules (Dall’Aglio et al. 2012). In particular, the presence of an evident immunopositivity for APLN also in the cytoplasm of endothelial cells could allow us to hypothesize that in queen’s placenta, locally produced APLN could carry out its actions both in autocrine/paracrine and endocrine ways. For this reason, we can say that our results could provide important information for understanding the role of APLN on placenta physiology.

In contrast to healthy pregnancies, Qi et al. (2025) discovered that individuals with early-onset severe preeclampsia had decreased levels of APLN expression in their placental tissue. Specifically, they demonstrated that the APLN/AKT/mTOR pathway was implicated in the downregulation of collagen type VI alpha 1 in the placenta affected by pre-eclampsia as compared to normal pregnancy.

Moreover, preeclampsia-related oxidative stress-induced placental dysfunction is related to decreased placental APLN expression, and elevated circulating APLN may be a somewhat effective marker to distinguish pre-eclamptic subjects from healthy pregnant women. Potential molecular mechanisms by which apelin-13/APLN shields placental trophoblasts from oxidative stress injury include upregulating superoxide dismutase activity/expression, inhibiting both superoxide production, and caspase-3 cleavage (Ye et al. 2023).

Even if it is a different species, even in queens the action of APLN at the placental level could be related to disorders in pregnancy. In future studies we will be able to analyze the action of APLN in queens and the pathways involved.

In conclusion, the ability of the apelinergic system to affect placenta cell proliferation in addition to its neoangiogenic properties lets us consider this system as a new important factor in assuring the physiological development of placenta, embryo and foetal growth in humans and small animals.

This descriptive study lays the foundation for future studies, in which we will investigate the role of the apelinergic system in the feline placenta through quantitative analyses.

## Data Availability

No datasets were generated or analysed during the current study.
